# Sentinel surveillance for enterovirus 71, Taiwan, 1998.

**DOI:** 10.3201/eid0503.990321

**Published:** 1999

**Authors:** T N Wu, S F Tsai, S F Li, T F Lee, T M Huang, M L Wang, K H Hsu, C Y Shen

**Affiliations:** Disease Surveillance and Quarantine Service, Ministry of Health, Taiwan, Republic of China.

## Abstract

Outbreaks of enterovirus 71 have been reported around the world since 1969. The most recent outbreak occurred in Taiwan during April-July 1998. This hand, foot, and mouth disease epidemic was detected by a sentinel surveillance system in April at the beginning of the outbreak, and the public was alerted.


### Sentinel Surveillance for Enterovirus 71, Taiwan, 1998

**Trong-Neng Wu,* Su-Fen Tsai,* Shu-Fang Li,* Tsuey-Fong Lee,* Tzu-Mei Huang,* Mei-Li Wang,* Kwo-Hsiung Hsu,† and Chen-Yang Shen‡**
*Disease Surveillance and Quarantine Service, Ministry of Health, Taiwan, Republic of China; †Bureau of Communicable Disease Control, Ministry of Health, Taiwan, Republic of China; and ‡Institute of Biomedical Sciences, Academia Sinica, Taipei, Taiwan, Republic of China

Outbreaks of enterovirus 71 have been reported around the world since 1969. The most recent outbreak occurred in Taiwan during April-July 1998. This hand, foot, and mouth disease epidemic was detected by a sentinel surveillance system in April at the beginning of the outbreak, and the public was alerted.

Enterovirus type 71 (EV71), one of the etiologic agents of epidemic hand, foot, and mouth disease (HFMD), has been associated with febrile rash illness, aseptic meningitis, encephalitis, and a syndrome of acute flaccid paralysis similar to that caused by poliovirus (1,2). EV71 was identified in 1969 in the United States, when it was isolated from the feces of an infant with encephalitis in California. By 1998, many EV71 outbreaks had been reported around the world. In addition to the outbreak in California in which one death was reported, four other outbreaks resulted in many fatal cases involving clinical deterioration and death in young children (Bulgaria, May-September 1975; Hungary, 1978; Malaysia, April-June 1997; Taiwan, April-August 1998) (3-5). To the best of our knowledge, the outbreak in Taiwan marked the first time that an EV71 outbreak was detected by a surveillance system, which alerted the public about the epidemic of HFMD. We describe how the EV71 outbreak was reported by a sentinel surveillance system established in July 1989 by Disease Surveillance and Quarantine Service (originally National Quarantine Services), Ministry of Health, Taiwan.

In this sentinel surveillance system, public health officers contact local and regional physicians weekly to actively collect disease information. On the basis of information collected, disease incidence trends are predicted, and, if necessary, the public is warned during the earliest stage of disease outbreaks. Mumps, varicella, diarrhea, and upper-tract respiratory infection are among the diseases subject to routine surveillance. For convenience, we have established two channels of data collection: telephone interviews and report cards mailed by physicians. Approximately 850 physicians (fewer than one tenth of the physicians) from every county in Taiwan participate in the system: 258 in the northern region, 211 in the central region, 296 in the southern region, and 85 in the eastern region (Table); most of these are pediatricians, general practitioners, and family physicians. Only a few are ear, nose, and throat specialists.

When an epidemic of fatal myocarditis was reported in Sarawak, Malaysia, in 1997, we started to collect information regarding HFMD and vesicular pharyngitis (herpangina) through

Table. Physician distribution, Sentinel Surveillance System, Taiwan

| Region of Taiwan | Interviews | No. cards mailed by physicians | Total no. physicians |
|---|---|---|---|
| Northern | 109 | 149 | 258 |
| Central | 27 | 184 | 211 |
| Southern | 175 | 121 | 296 |
| Eastern | 13 | 72 | 85 |
| Total | 324 | 526 | 850 |

Address for correspondence: Su-Fen Tsai, Division of Disease Surveillance, Disease Surveillance and Quarantine Service, Ministry of Health, F6, No.6, Lin Shen S. Rd., Taipei, Taiwan, R.O.C.; fax: 886-2-23945312; e-mail: sftsai@net.dsqs.gov.tw.

the sentinel surveillance system. Beginning in March 1998, some physicians reported a notable increase of cases of HFMD, vesicular pharyngitis (herpangina), and vesicular stomatitis exanthem. Furthermore, a dramatic upsurge of HFMD was seen in children in outpatient settings at the end of April 1998 (Figure 1).

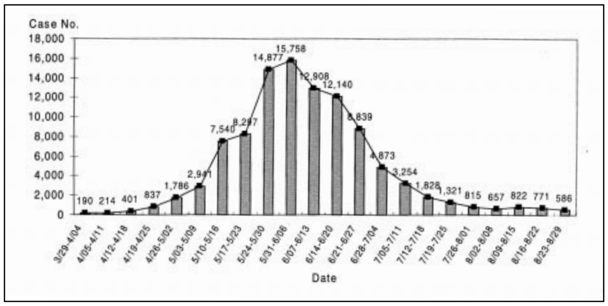

Figure 1. Total cases of hand, foot, and mouth disease and herpangina reported from sentinel physicians in Taiwan, March 19 to August 29, 1998.

Surveillance data provided the basis for immediate public health action. When the number of weekly reported cases increased twofold at the end of April, the public was informed about the epidemic of HFMD and herpangina and the threat of enterovirus infection (May 12). Measures for preventing the spread of infection (e.g., practicing good hygiene at all times, confining infected children at home, avoiding contact with infected children) were advised. However, the number of reported cases still dramatically increased to approximately 3,000 in the following week. Although some errors in reporting might have occurred, we are confident that the reporting quality was adequate. The sentinel physicians, who have voluntarily cooperated with us for 10 years, used a clinical case definition of HFMD/herpangina we created when the surveillance started.

Monitoring incidence of the fatal and most severe cases of HFMD appeared critical; therefore, another report system, designed for monitoring severe and fatal cases, was established (May 29) to enroll all well-defined severe and fatal cases in 597 hospitals and medical centers. This new system established a network of various public health agencies, general and regional hospitals, and medical centers. The difference between the two systems was that, while the sentinel surveillance system was physician-based, the new system was hospital-based. Severe and fatal cases were defined as HFMD with complications, including aseptic meningitis, encephalitis, myocarditis, acute flaccid paralysis, rapidly deteriorating clinical course, and death. Both surveillance systems worked simultaneously from June 1998 onward. We found that the trend peaked and declined earlier in the sentinel system than in the hospital-based surveillance system (Figure 1, 2).

A case-control study was implemented, and enterovirus isolation data were reviewed. Several enterovirus isolations, from patients with severe and fatal cases, were in stool specimens, throat secretions, cerebrospinal fluid, blood, and central nervous system tissue, including EV71, Coxsackie, and ECHO. Additional studies comparing rates of EV71 isolation in different years are in progress and will be reported separately. Most isolated viruses were EV71 from all specimens in this epidemic. The epidemiologic, clinical, and virologic evidence suggests an association between EV71 infection and this epidemic of HFMD. However, the causes of the severe cases and deaths in Taiwan are yet to be defined (5).

A physician-based sentinel surveillance system can play an important role in preventing emerging infectious diseases. Even though the data collected may be rough, a sentinel surveillance system can provide necessary information for monitoring communicable diseases, guiding further investigation, and evaluating control measures, as well as early warning for epidemics and rationale for public

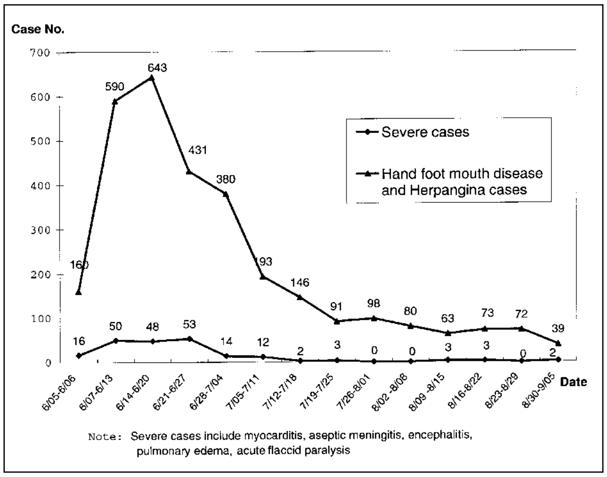

Figure 2. Number of hospitalizations and severe cases of hand, foot, and mouth disease and herpangina in Taiwan, June-August, 1998.

health intervention. Early detection of communicable diseases and immediate public health intervention can curtail the number of illnesses and deaths and reduce negative effects on international travel and trade (6).

Dr. Wu is vice superintendent of Municipal Hsiao-Kang Hospital, Kaohsiung Medical College, Taiwan, Republic of China. He is also a professor at both Kaohsiung Medical College, Kaosiung, Taiwan, and China Medical College, Taichung, Taiwan.

### References

1. Melnick JL. Enterovirus type 71 infection: a varied clinical pattern sometimes mimicking paralytic poliomyelitis. Rev Infect Dis 1984;6 Suppl:S387-90.
2. Alexander JP Jr, Baden L, Pallansch MA, Anderson LJ. Enterovirus 71 infection and neurologic disease-United States, 1977-1991. J Infect Dis 1994;169:905-8.
3. Shindarov LM, Chumakov MP, Voroshilova MK, Bojinov S, Vasilenko SM, Iordanov I, et al. Epidemiological, clinical, and pathomorphological characteristics of epidemic poliomyelitis-like disease caused by enterovirus 71. Journal of Hygiene, Epidemiology, Microbiology, and Immunology 1979;23:284-95.
4. Nagy G, Takatsy S, Kukan E, Mihaly I, Domok I. Virological diagnosis of enterovirus type 71 infections: experiences gained during an epidemic of acute CNS diseases in Hungary in 1978. Arch Virol 1982;71:217-27.
5. Centers for Disease Control and Prevenetion. Deaths among children during an outbreak of hand, foot, and mouth disease–Taiwan, Republic of China, April-July 1998. MMWR Morb Mortal Wkly Rep 1998;47:629-32.
6. Heymann DL, Rodier GR. Global surveillance of communicable diseases. Emerg Infect Dis 1998;4:362-5.

